# Evaluation of radiation dose to organs during kilovoltage cone‐beam computed tomography using Monte Carlo simulation

**DOI:** 10.1120/jacmp.v15i2.4556

**Published:** 2014-03-06

**Authors:** Kihong Son, Seungryong Cho, Jin Sung Kim, Youngyih Han, Sang Gyu Ju, Doo Ho Choi

**Affiliations:** ^1^ Department of Nuclear and Quantum Engineering Korea Advanced Institute of Science and Technology Daejeon Korea; ^2^ Department of Radiation Oncology Samsung Medical Center, Sungkyunkwan University School of Medicine Seoul Korea

**Keywords:** GATE, XCAT, CBCT, absorbed dose

## Abstract

Image‐guided techniques for radiation therapy have improved the precision of radiation delivery by sparing normal tissues. Cone‐beam computed tomography (CBCT) has emerged as a key technique for patient positioning and target localization in radiotherapy. Here, we investigated the imaging radiation dose delivered to radiosensitive organs of a patient during CBCT scan. The 4D extended cardiac‐torso (XCAT) phantom and Geant4 Application for Tomographic Emission (GATE) Monte Carlo (MC) simulation tool were used for the study. A computed tomography dose index (CTDI) standard polymethyl methacrylate (PMMA) phantom was used to validate the MC‐based dosimetric evaluation. We implemented an MC model of a clinical on‐board imager integrated with the Trilogy accelerator. The MC model's accuracy was validated by comparing its weighted CTDI (CTDIw) values with those of previous studies, which revealed good agreement. We calculated the absorbed doses of various human organs at different treatment sites such as the head‐and‐neck, chest, abdomen, and pelvis regions, in both standard CBCT scan mode (125 kVp, 80 mA, and 25 ms) and low‐dose scan mode (125 kVp, 40 mA, and 10 ms). In the former mode, the average absorbed doses of the organs in the head and neck and chest regions ranged 4.09‐8.28 cGy, whereas those of the organs in the abdomen and pelvis regions were 4.30‐7.48 cGy. In the latter mode, the absorbed doses of the organs in the head and neck and chest regions ranged 1.61‐1.89 cGy, whereas those of the organs in the abdomen and pelvis region ranged between 0.79‐1.85 cGy. The reduction in the radiation dose in the low‐dose mode compared to the standard mode was about 20%, which is in good agreement with previous reports. We opine that the findings of this study would significantly facilitate decisions regarding the administration of extra imaging doses to radiosensitive organs.

PACS number: 87.57.uq

## I. INTRODUCTION

X‐ray imaging is currently an important method for patient positioning, target localization, and external beam adjustment in radiation therapy. Moreover, it has contributed to significant progress in image‐guided radiation therapy (IGRT),^1^, ^2^, ^3^ which has greatly improved the precision of radiation delivery and the sparing of normal tissues.^4^, ^5^, ^6^ Kilovoltage (kV) cone‐beam computed tomography (CBCT)^(7)^ is a widely used IGRT technique owing to its rich image information and faster and more robust image acquisition process. High‐precision radiotherapy is critical when the surrounding tissues are highly radiosensitive.^(8)^ Daily CBCT particularly enables the acquisition of accurate three‐dimensional information about the anatomy of a patient for every treatment fraction, and is also suitable for planning adaptive radiotherapy treatment.^5^, ^9^, ^10^, ^11^ It may, however, increase health risks associated with imaging radiation. Accurate dosimetry of imaging radiation from CBCT scans is, therefore, important because it would provide radiation oncologists with useful information about excessive doses to radiosensitive organs.^(5)^ Unlike general diagnostic imaging and image‐guided surgery, IGRT adds an imaging dose to the therapeutic radiation dose, which is already high.^(1)^ This could create a complex distribution of the dose and increase the risk of the development of secondary malignancies, which has given rise to the need for ways of evaluating and minimizing the imaging dose.^(12)^ Accurate evaluation of the dose delivered to specific organs is very important to assessing the risk of complications due to CBCT scans. Previously developed dosimetric methods for diagnostic imaging include weighted computed tomography dose index (CTDIw), computed tomography dose index volume (${\text{CTDI}}_{\text{vol}}$), and dose‐length product (DLP). However, these methods produce a representative value of the radiation dose and not that of individual organs. This work was therefore aimed at investigating the imaging radiation dose delivered to various organs of a patient (including radiosensitive organs) using the 4D extended cardiac‐torso (XCAT) phantom^(13)^ and the Geant4 Application for Tomographic Emission (GATE) Monte Carlo (MC) simulation toolkit.^(14)^

## II. MATERIALS AND METHODS

### A. Monte Carlo simulation for CBCT geometry and CTDI validation

**Figure 1:**
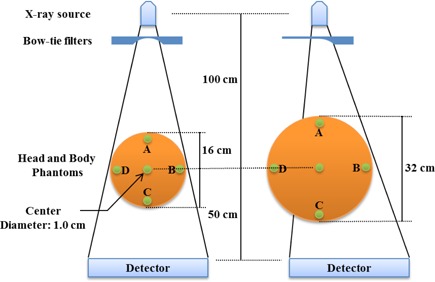
CTDIw geometry in which doses were calculated using GATE at five points (A, B, C, D, and center) in each of the head and body phantoms.

GATE is an MC simulation platform developed by the Open GATE collaboration in 2001 and was publicly released in 2004. The platform was initially specifically used for modeling planar scintigraphy, single‐photon emission computed tomography (SPECT), and positron emission tomography (PET) data acquisition.^15^, ^16^ More recently, it has also been used to model X‐ray computed tomography (CT) scanners.^(14)^ The simulation of the CBCT beams in this study utilizes an on‐board imager (OBI) (Varian Medical Systems, Palo Alto, CA) system integrated with a Trilogy linear accelerator (Varian Medical Systems). The entire OBI CBCT system, which includes the X‐ray tube, beam defining system, the phantom, and the flat‐panel detector, was simulated.^(17)^ The X‐ray spectrum was obtained from the Institute of Physics and Engineering in Medicine (IPEM) Report 78.^(18)^ A typical X‐ray energy spectrum was generated from a tungsten target at 125 kVp after Al filtering. The beam quality was validated by comparing the point doses with published CTDIw values.^(6)^ The CBCT dose was measured using standard PMMA head and body CTDI phantoms (Radical Computing Corporation, Rocky Hill, CT) of height 15 cm and diameters 16 and 32 cm, respectively. Nine holes were in each of the head and body phantoms, which could accommodate an ion chamber or acrylic dummy plug. The CBCT dose was evaluated in the five holes (A, B, C, D, and the center), as shown in Fig. 1. The distance between the center and each of the holes was 7 cm in the head phantom and 15 cm in the body phantom.^(19)^

### B. XCAT phantom

Recent advances in computerized phantom have resulted in the development of an ideal model that integrates a realistic patient‐based voxelized phantom with a mathematical or stylized phantom. The XCAT phantom, for example, provides a realistic and flexible model of the entire human body for the study of high‐resolution medical imaging such as CT, PET, and SPECT.^13^, ^20^ In this work, the voxelized XCAT phantom was converted into an interfile format for use in the GATE toolkit. The interfile format was used to classify the anatomical components based on their voxel values or CT numbers. The CT numbers were then converted into electron density using a Geant4 materials library file. For this purpose, a user‐defined mass density tolerance was used to establish piecewise linear correspondence between the CT numbers and mass densities. A list of material compositions was referenced for the conversion. The generated list of materials and the correspondence between the materials and CT numbers were then stored for use in converting any CT image into component materials.^(14)^ The number of CT slices considered in evaluating the dose for imaging the head and neck, chest, abdomen, and pelvis sites were 120, 95, 160, and 65, respectively. A constant slice thickness of 2.5 mm was used and each slice had an image array of 512×512 and pixel pitch of $0.84\times 0.84\;{\text{mm}}^{2}$. We estimated the doses administered to various organs such as the skin, cord, brain, eye, lung, heart, liver, intestine, kidney, prostate, bladder, ovary, uterus, and rectum. The evaluated organ doses represented the imaging radiation dose received by a patient during kV CBCT acquisition at the corresponding site.

### C. CBCT geometry and parameters

**Figure 2:**
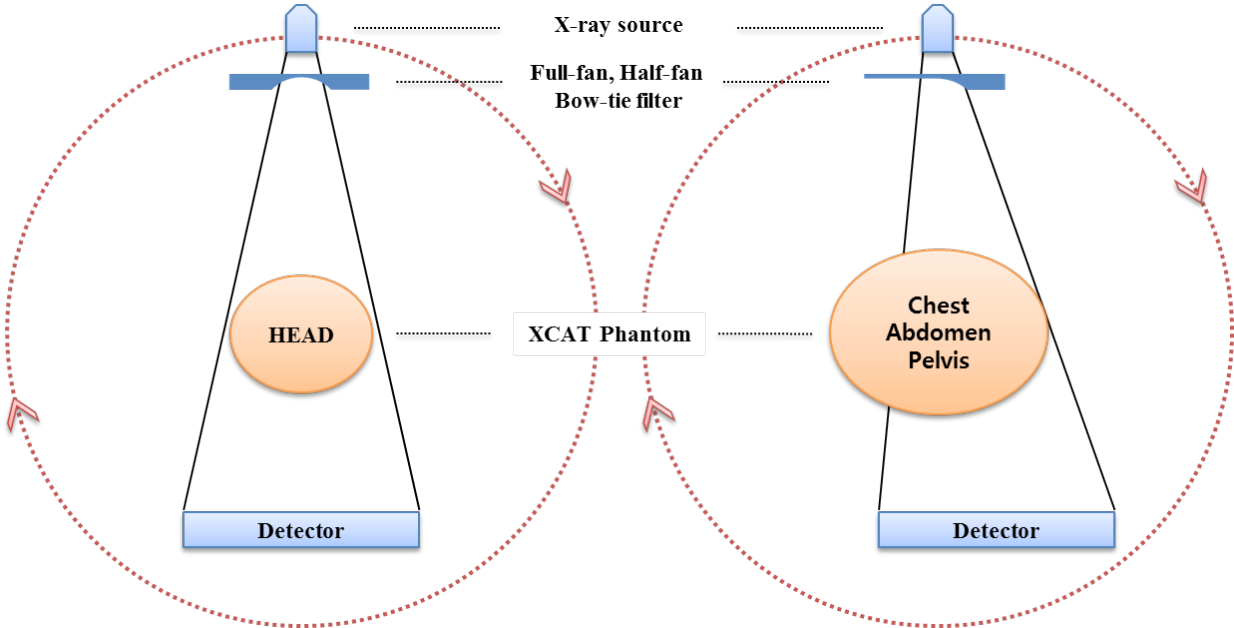
Scheme of simulated scanning geometry in which XCAT phantom and two types of bowtie filters (full‐fan and half‐fan) were used.

The OBI‐CBCT system incorporates an amorphous silicon (aSi) flat‐panel detector with a 2048×1536 array of 0.194 mm elements. In this study, we used a fixed source‐to‐isocenter distance of 100 cm and source‐to‐detector distance of 150 cm. The CBCT projections can be acquired in two modes, namely full‐fan and half‐fan modes. The manufacturer of the OBI device recommends the use of an additional filter (the bowtie) for improved image quality during CBCT acquisition. We used two types of bowtie filters, namely the full‐fan and the half‐fan bowtie. The two bowtie filters were designed for use in the full‐fan and half‐fan acquisition modes, respectively. Schematics of the scanning geometries for the full‐fan and half‐fan scanning modes are shown in Fig. 2. The default CBCT beam conditions of 125 kVp, 80 mA, and 25 ms were used for the standard scanning mode, whereas 125 kVp, 40 mA, and 10 ms were used for the low‐dose scanning mode. Although the low‐dose scanning mode produces less imaging dose and lower quality images,^(5)^ Kan et al.^(21)^ systematically compared the contrast, spatial resolution, and noise level of the images acquired in the two scanning modes and reported that the difference between the image qualities was not significant and did not affect the patient localization process. The full‐fan mode was specifically used for the head region because of its narrow field of view (FOV), whereas the half‐fan mode was used for other sites such as the chest, abdomen, and pelvis. The scanning parameters are summarized in Table 1.

**Table 1:** CBCT scanning parameters for standard dose and low‐dose modes

*Parameter*	*Standard Mode*	*Low Dose Mode*
Voltage (kVp)	125	125
Current (mA)	80	40
Millisecond (ms)	25	10
Rotation angle (°)	360	360
Bowtie filter	full/half‐fan	full/half‐fan
Direction	clockwise	clockwise

## III. RESULTS

### A. Validation of the MC model

Five dosimetric points in the CTDI phantoms were used for the validation experiment and the doses were found to range between 0.76 and 9.12 cGy (see Table 2). The measurements were used to calculate the CTDIw values, which were found to be 8.90 and 4.78 cGy for full‐fan and half‐fan in the standard scanning mode, respectively. The corresponding CTDIw values in the low‐dose scanning mode were, respectively, 2.04 and 1.09 cGy. The differences between the CTDIw values obtained in this study and those reported for the MC calculations conducted by Kim et al.^(6)^ were between 0.07 and 0.19 cGy for the head and body phantoms, indicating good agreement. The dose distribution between the peripheral and central points was more uniform in the full‐fan mode.

**Table 2:** Comparison of MC simulation results for standard dose and low‐dose modes

	Head Phantom (=Full−fan)		Body Phantom (=Half−fan)	
*Locations*	*Standard Mode*	*Low Dose Mode*	*Standard Mode*	*Low Dose Mode*
Center	8.83±0.12	1.98±0.02	3.50±0.07	1.76±0.02
A	8.96±0.14	2.04±0.03	5.54±0.08	1.25±0.03
B	9.12±0.13	1.08±0.01	5.34±0.06	1.26±0.01
C	9.15±0.02	2.10±0.03	5.36±0.07	1.21±0.02
D	9.09±0.14	2.03±0.02	5.43±0.08	1.30±0.03
Avg. of peripheries	9.05	2.06	5.42	1.25
CTDIw	8.90	2.04	4.78	1.09
CTDIw^(6)^	8.76	1.96	4.65	1.16
Differences	0.14	0.08	0.13	−0.07

Unit=cGy.

### B. Absorbed doses of organs

The radiation doses delivered to the organs during the OBI scans are summarized in Table 3. In the standard CBCT scan mode (125 kVp, 80 mA, and 25 ms), the calculated organ doses ranged between 4.30 and 8.28 cGy. For the head and neck scan, we calculated the organ doses delivered to the eyeball, brain, cord, and soft tissue, including the skin. The eyeball received 7.38 cGy, the brain 7.92 cGy, the spinal cord 8.28 cGy, and the soft tissue 3.80 cGy. For the chest scan, we calculated the doses delivered to the lung, heart, spinal cord, and soft tissue, including the skin. The right and left lungs received 6.10 and 7.48 cGy, respectively; the heart received 6.33 cGy; and the spinal cord and soft tissue received 4.09 and 6.33 cGy, respectively. For the abdomen scan, we evaluated the doses delivered to the liver, intestine, and kidney. The liver was determined to have received 6.15 cGy; the intestine received 7.40 cGy; and the left and right kidneys received 7.48 and 6.10 cGy, respectively. For the pelvis scan, the bladder, prostate, rectum, ovary, and uterus received 5.57, 5.37, 4.30, 4.46, and 4.47 cGy, respectively. We also calculated the organ doses delivered in the low‐dose mode. The corresponding results are summarized in Table 3. The delivered doses of the head and neck scan ranged between 1.61 and 1.78 cGy, and those of the other sites ranged between 0.79 and 1.85 cGy.

**Table 3:** Absorbed doses of organs in standard dose and low‐dose modes for OBI‐CBCT system. All units are cGy

		*Standard Mode Scan 125 kV, 80 mA, 25 ms*					
*Full‐fan Bowtie Filter Head and Neck*		*Chest*		*Half‐fan Bowtie Filter Abdomen*		*Pelvis*	
Eyeball	7.38	Rt. Lung	6.58	Liver	6.15	Bladder	5.57
Brain	7.92	Lt. Lung	6.19	Intestine	7.40	Prostate	5.37
Cord	8.28	Heart	4.09	Rt. Kidney	6.10	Rectum	4.30
Soft tissue	7.59	Cord	5.90	Lt. Kidney	7.48	Ovary	4.46
		Soft Tissue	6.50			Uterus	4.47
		*Low‐Dose Mode Scan 125 kV, 40 mA, 10 ms*					
*Full‐fan Bowtie Filter Head and Neck*		*Chest*		*Half‐fan Bowtie Filter Abdomen*		*Pelvis*	
Eyeball	1.61	Rt. Lung	1.48	Liver	1.44	Bladder	1.47
Brain	1.89	Lt. Lung	0.87	Intestine	1.78	Prostate	1.17
Cord	1.85	Heart	1.46	Rt. Kidney	1.85	Rectum	1.27
Soft tissue	1.78	Cord	1.34	Lt. Kidney	1.52	Ovary	1.09
		Soft Tissue	1.53			Uterus	0.79

## IV. DISCUSSION

An accurate assessment of the imaging radiation doses of CBCT scans is important considering their widespread application in linac‐mounted or stand‐alone CBCT systems used for image‐guided radiation therapy.^(22)^ Kim et al.^6^, ^22^ developed an MC model of an OBI‐CBCT system based on BEAMnrc/EGSnrc, and validated the beam performance by comparing the half‐value layer (HVL) and X‐ray spectrum data with those reported by Ding et al.^(17)^ and those published in the IPEM Report 78. Kim and colleagues also evaluated the CTDIw values of CBCT using MOSFET detectors, and incorporated the MC model of both the head and body phantoms in the simulation. Their results were comparable with those of Song et al.^(23)^ In this study, we compared the point doses and CTDIw values with the data reported by Kim et al.^(6)^ to validate the proposed GATE‐based dosimetric calculation method. As shown in Table 2, the measured dose in the standard scanning mode agreed very well with those of the Kim study to within 1.6% and 2.8% for the head and body phantoms, respectively. Our evaluated doses for the head and body phantoms in the low‐dose scanning mode were also in agreement to within 4.1% and 6.1%, respectively.

We postulate that the increased discrepancies in the low‐dose mode were due to the smaller number of X‐ray photons. Regarding the dose distribution between the periphery and the center, the pattern of the head scan was more uniform than that in the body scan. The body phantom dose distribution at the periphery was about 1.5 to 1.7 times that at the center. A comparison of our data with those of the Song^(23)^ and Kim^(6)^ studies revealed similar tendencies. This is considered to be mainly due to the mean attenuation path length of the central point of the CTDI body phantom being longer than that of the peripheral points. The mean attenuation path lengths are, however, similar for the head phantom.^(24)^ Furthermore, the differences between our CTDIw values and those provided by the vendor for the standard and low‐dose modes were less than 1 cGy.^(25)^

Based on this validation, we extended our study to the XCAT phantom to evaluate the specific organ dose. As described earlier, GATE was used to calculate the doses to various sites including the head‐and‐neck, chest, abdomen, and pelvis regions. We compared the calculated organ doses with the data of Ding et al.,^4^, ^5^, ^26^ who used the Vanderbilt‐Monte‐Carlo‐Beam‐Calibration (VMCBC) (Vanderbilt University, Nashville, TN) algorithm to calculate the radiation doses administered to organs in full‐fan and half‐fan kV CBCT scan modes. The voxel size of the phantom was $0.25\times 0.25\times 0.25\;{\text{cm}}^{3}$. The results showed that the organ doses in the head and neck, chest, and abdominal regions during a single CBCT procedure ranged between 1 and 9 cGy for the soft tissues, and between 6 and 29 cGy for the bones.

Although the organ doses in the standard mode evaluated in our study were generally in good agreement with those reported in the studies by Ding and colleagues, there were relatively significant differences between the doses absorbed by the brain, prostate, and the cord. The values of our study were consistently 2 to 3 cGy higher, which we believe was due to the different types and sizes of the phantoms used for the two studies. The voxelized phantom used by Ding et al.^5^, ^26^ was produced by adapting a real patient CT image, and the mass densities and sizes of the organs might have been substantially different from those of the XCAT phantom. Winey et al.^(27)^ reported kV CBCT dose measurements in the standard scanning mode using a half‐fan filter. A 0.65 cm^3^ Farmer type ionization chamber was used to obtain the absorbed dose of the lung and skin in an anthropomorphic phantom. The measured doses were 4.59, 5.16, and 5.31 cGy for the right lung, left lung, and skin, respectively. These values were 1‐2 cGy lower than ours, which is believed to be due to the different scanning conditions and phantoms. In the low‐dose mode, the absorbed doses were approximately 20% of those absorbed in the standard mode, which agrees well with the report of Kan et al.^(21)^ The Kan study used a female anthropomorphic phantom containing thermoluminescent diodes (TLDs) and observed that the radiation doses were reduced by 20%.

The organ dose administered by a daily CBCT scan can be significant, and the additional imaging radiation poses an increased risk to radiosensitive organs.^(5)^ Although the imaging dose is much lower than the radiation dose typically administered in radiotherapy, it requires special attention. This is because the dose distribution of the former is quite different from that of a therapeutic beam and the effects of X‐ray photons on radiosensitive organs cannot be neglected. This is particularly important when fractionated radiation therapy is used for daily imaging.

## IV. CONCLUSIONS

We used the GATE toolkit to simulate kV CBCT scans and used an XCAT phantom to calculate the radiation doses delivered to various organs. We demonstrated the validity of the method by comparing its CTDIw values with those of previous reports, which revealed good agreement. The proposed method has various potential uses, especially for dosimetric assessment in imaging. It is our expectation that this tool kit would be useful to clinicians for immediate onsite determination of the imaging radiation dose administered to various organs, particularly during daily image‐guided IGRT. This would facilitate decisions regarding the administration of additional imaging doses to radiosensitive organs.

## ACKNOWLEDGMENTS

This research was supported by Basic Science Research Program through the National Research Foundation of Korea (NRF) funded by the Ministry of Education, Science and Technology (NRF‐2011‐0022021), Radiation Safety Programs (2011‐31115) through the NSSC, the Technology Innovation Program, 10040362 by the Ministry of Knowledge Economy (MKE, Korea), and Samsung Medical Center grant [GFO1130081]. K. Son and S. Cho were partly supported by NRF‐2013K000093 and MEST grant R0001270.
